# The efficiency of a new automated mosquito larval counter and its impact on larval survival

**DOI:** 10.1038/s41598-019-43333-0

**Published:** 2019-05-15

**Authors:** W. Mamai, H. Maiga, M. Gárdos, P. Bán, N. S. Bimbilé Somda, A. Konczal, T. Wallner, A. Parker, F. Balestrino, H. Yamada, J. R. L. Gilles, J. Bouyer

**Affiliations:** 1 Insect Pest Control Laboratory, Joint FAO/IAEA Division of Nuclear Techniques in Food and Agriculture, Vienna, Austria; 2 Institut de Recherche Agricole pour le Développement (IRAD), Yaoundé, Cameroon; 3 Institut de Recherche en Sciences de la Santé/Direction Régionale de l’Ouest (IRSS/DRO), Bobo-Dioulasso, Burkina Faso; 4 Radiation General Ltd. 1118 Budapest, Sasadi út 36, Budapest, Hungary; 5 Medical and Veterinary Entomology Department, Centro Agricoltura Ambiente CAA “G. Nicoli”, Crevalcore, Italy

**Keywords:** Entomology, Biological physics

## Abstract

To achieve consistent and standardized rearing for mosquito immature stages, it is crucial to control the initial number of larvae present in each larval tray. In addition, maintaining an optimal and synchronized development rate of larvae is essential to maximize the pupal production and optimize male sorting in a mass-rearing setting. Manual counting is labor intensive, time consuming and error prone. Therefore, this study aimed to investigate the use of a customized automated counter for the quantification of mosquito larvae. The present prototype of the mosquito larval counter uses a single counting channel consisting of three parts: a larvae dispenser, an electronic counting unit and computer control software. After the separation of the larvae from eggs and debris, batches of different numbers of *Aedes aegypti* first instar larvae were manually counted and introduced into the counter through the upper loading funnel and channeled out from the bottom of the counter by gravitational flow. The accuracy and repeatability of the mosquito larval counter were determined in relation to larval density and water quality. We also investigated its impact on larval survival. Results showed an impact of larval density and water quality on the accuracy of the device. A −6% error and a repeatability of +/− 2.56% average value were achieved with larval densities up to 10 larvae/mL of clean water. Moreover, the use of the mosquito larval counter did not have any effect on larval survival or development. Under recommended conditions, the mosquito larval counter can be used to enumerate the number of mosquito larvae at a given density. However, future developments involving the use of multiple channels or larger input larvae container would help to expand its use in large-scale facilities.

## Introduction

Mosquitoes are insects broadly distributed around the world, particularly in tropical and sub-tropical regions. Various mosquito species, among them *Anopheles*, *Aedes*, and *Culex*, include vectors of many viruses and parasites that affect humans with illnesses such as malaria, dengue fever, yellow fever, Chikunguya, West Nile fever, and Zika. Vector control is an important component of many disease control programmes. Therefore, research on these mosquitoes is crucial for the development of control strategies that target transmission by the vectors. Mosquito research laboratories around the world usually require rearing of mosquitoes for a wide variety of purposes including studies in vector biology, physiology, vector-parasite interactions, insecticide susceptibility and behavior. A careful balance of larval-numbers with water surface, water volume and diet is essential for survival and uniform, optimal growth and development of mosquito larvae reared for experimental purposes^1,2^. Accurate estimation of the number of first instar larvae is, therefore, central to controlling the rearing density. Thus, early stage (eggs, first instar larvae) quantification constitutes an essential task performed by laboratory technicians for routine and research activities. The initial number of larvae present in each rearing tray should be controlled to accurately measure or estimate the effect of any factor on mosquito development or life history traits. Such numbers are sometimes over 500 larvae per rearing tray in small scale. In the context of the sterile insect technique (SIT), accurate estimates of the initial number of larvae are of utmost importance to ensure synchronized larval development, maximize the pupal productivity and optimize male sorting in a mass-rearing facility. Further, fertility is based on counts of first instar larvae and is one of the most important parameters to determine radiation induced sterility and competitiveness of sterile males^3^.

Currently, quantification methods used to estimate the number of larvae relies on manually counting them, whereas weight and/or volume can be used for the quantification of eggs^4^. Although manual counting (considered as a gold standard reference method) is simple, it is labor intensive, time consuming, causes visual fatigue, requires man power and a high level of attention by the performer. Most often it is difficult to count the total number of larvae needed for a specific purpose at one time. Hence, there remains a need for an easy tool which reduces time and resources required to perform a variety of assays. Developing an automated system has become a necessity and would offer faster, accurate and more reproducible results. Moreover, implementation of an automated counting system would eliminate the subjectivity of manual counting and minimize user-to-user variability.

Several earlier studies have produced programs that enumerate mosquitoes, mites and malaria parasites^5–8^. However, automatic counting of mosquito larvae is a subject that has received little attention. A microcontroller based dedicated electronic counting device has been designed and developed by Jahan *et al*.^9^, for their research which requires the counting of late instar mosquito larvae and pupae. However, this device which can only count up to 999 mosquitoes is not available to buy. Moreover, a mosquito larval counter is currently being developed by Orinno Technology in Singapore that can be used to count mosquito larvae^10^.

The mosquito larval counter (MLC) single channel (CH1) model (hereafter MLC-CH1 v1.0) is an automated system designed and developed in collaboration with Radiation General Ltd (RadGen), Budapest, Hungary to count immature stages of the mosquitoes, including L1s, as they pass through the optical sensor unit. The optical sensor measures a physical quantity of light and then translates it into a form that is readable by an integrated measuring device. The transmitter (light source) projects a light beam onto the receiver. The object or larvae interrupts the light beam sent out by a light source connected to fibre optical sensor when it passes through. The interruption of the light beam is evaluated and interpreted as a switch signal by the receiver. The purpose of this study was to investigate the efficacy of the MLC-CH1 v1.0 to perform automated counting of first instar mosquito larvae with the ultimate goal of controlling the initial larval rearing density and therefore standardizing the overall rearing process. It is possible that inaccuracies can occur through factors such as overlapping larvae when they are crowded, differences in their size due to different species, different age and presence of particles in the water. Thus, factors which could affect the accuracy need to be controlled. Therefore, the objectives were to: (i) determine how closely a measure from the MLC-CH1v1.0 approximates its true value (reference to manual counting) (hereafter referred as accuracy); (ii) determine the closeness of multiple measurement values (repeatability), (iii) evaluate the effect of larval density on the accuracy, (iv) evaluate the possibility of using the MLC for other mosquito stages and species and finally (v) examine whether MLC treatment negatively affects survival and development of MLC counted larvae.

## Results

### Experiment 1: Accuracy of the mosquito larvae counter

In the preliminary trial using a larval density of 10 larvae/mL, the accuracy, expressed as percent error, ranged between −5.00 and −9.35% and the mean percent error was −7.14%. The negative number indicates that the MLC underestimated the number of larvae by 7.14% of its reference or manual count value.

**Figure 1:**
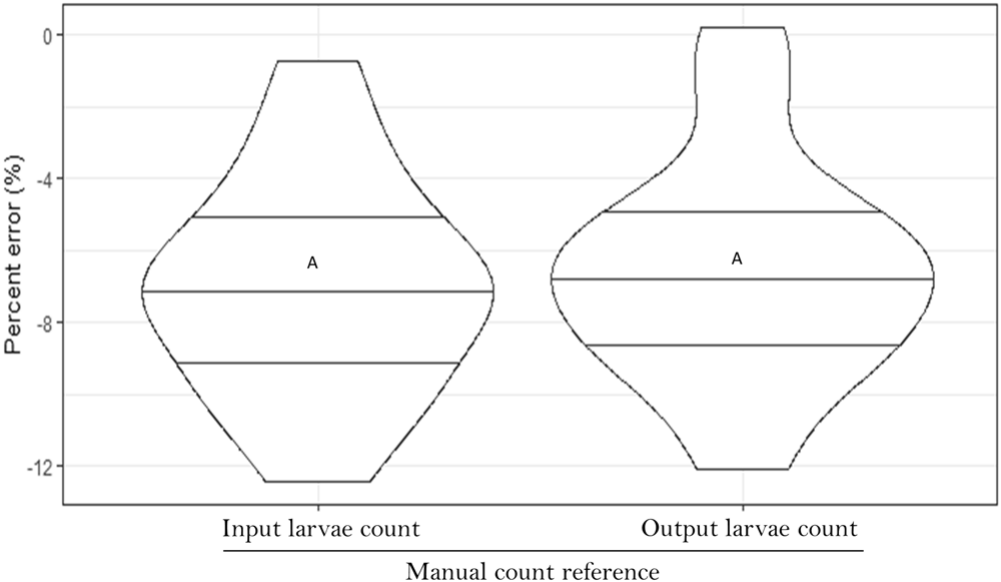
Relative accuracy (percent error) of the automated mosquito larval counter using first instar larvae in reference to the input and the output larvae manual count. Each violin plot is an average of 14 replicates or counts. Each violin box denotes the median as a line across the middle, the quartiles (25th and 75th percentiles), the minimum and maximum values at its ends. Different letters indicate statistically different results between treatments.

In the second trial, the mean percent error was −7.01 and −6.56% relative to input and output larvae count respectively (Fig. 1) with no significant difference between the manual input larvae count and the manual output larvae count (t-test, *P* = 0.47). Input larvae are larvae in the container counted prior to automatic counting while output larvae are those in the container collected and counted after automated process. The egg hatching-weighing method showed an error of −7.05% similar to that of automatic counting. However, the repeatability within measurements was less good in the egg hatching-weighing method (16.59% of the average value, than the automated counting (less than 3%) (see Table 1).

**Table 1:** Repeatability of the mosquito larval counter evaluated as a function of larval densities.

Number of 1^st^ instar	Larval density (L1s/mL)	Percent of average value (%)	Corresponding repeatability (%)
2000	5	2.92	97.08
4000	10	2.70	97.30
6000	15	2.06	97.94

### Experiment 2: Assessing the effect of larval density on the accuracy and repeatability

**Figure 2:**
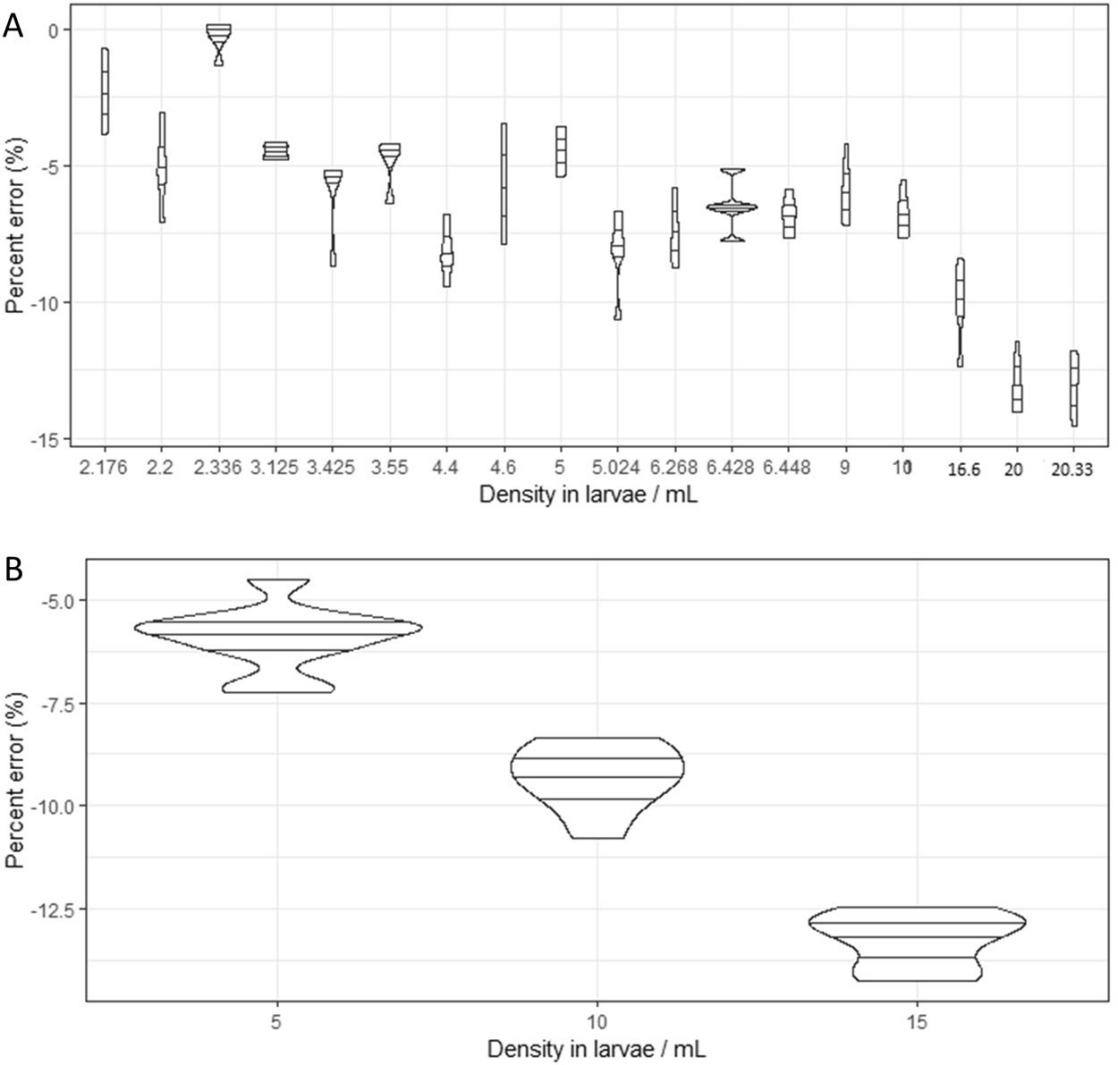
Relative accuracy (percent error) of the automated mosquito larval counter as a function of larval densities. Each violin box denotes the median as a line across the middle, the quartiles (25th and 75th percentiles), the minimum and maximum values at its ends. (**A**) First trial and (**B**) second trial.

In the preliminary trial, the larval density significantly influenced the accuracy of the counter (Fig. 2A, df = 81, t = −12.96, *P* < 0.001). With densities of larvae lower than 10 larvae/mL (range 2.2 to 10 larvae/mL), the MLC produced a count with an average of −2.53 to −7.14% percent error. However, with larval densities higher than 10 larvae/mL, the percent errors were markedly higher, with an average of −14.57%, indicating a low accuracy. With appropriate larval densities, the MLC presented the count values closest to the assumed reference value. The second trial using the densities of 5, 10 and 15 larvae/mL confirmed the trend with significant effect of larval density (Fig. 2B, df = 21, t = −24.45, *P* < 0.001).

### Within-run repeatability and time efficiency

In terms of repeatability, measurements were remarkably closer, deviating only 2.56% from the mean value. The percent of average values were 2.92, 2.70 and 2.06% corresponding to 97.08, 97.30 and 97.94% repeatability for the larval densities 5, 10, and 15 respectively; suggesting that the larval density did not alter the repeatability of the MLC (Table 1). The MLC was able to count larvae with a reasonable repeatability compared to a 83.41% repeatability of the manual counts.

Manually counting 1000 larvae took up to 30 minutes whereas the same number of larvae or more in a volume of 400 mL could be processed by the MLC in 1 minute and 23 seconds. The MLC took 83 seconds to drain 400 mL of water whatever the larval density.

### Experiment 3: Using the mosquito larval counter for different mosquito stages and ages

**Figure 3:**
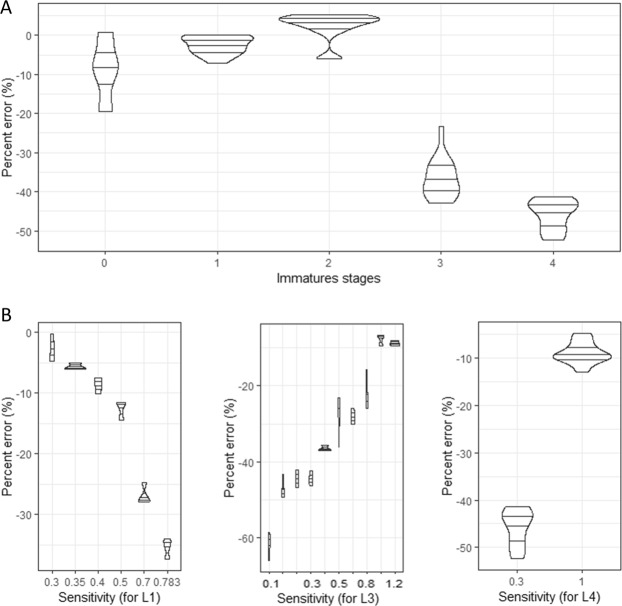
Relative accuracy (percent error) of the mosquito larval counter as a function of mosquito larval age for a calibration at the one-day-old larvae (**A**) and its variation following calibration for each mosquito larval age (**B**). L1, L3 and L4 represent one, three and four days-old larvae respectively.

Different mosquito stages and ages (eggs, larvae from one day to four day-old) were separately counted with the MLC using the same sensitivity value. Results showed that the accuracy significantly differed with mosquito stage (Fig. 3A, df = 58, t = 11.87, *P* < 0.001). Advanced stages (three and four day –old larvae) showed lower accuracies. The appropriate sensitivity value to obtain high accuracy varied with mosquito ages. For example, the appropriate sensitivity for first instar larvae (one day-old) was 0.300 and 1.00 for three day-old and four day-old larvae (Fig. 3B). Data indicated that users should perform prior calibrations for each mosquito age before counting.

### Experiment 4: Assessing the effect of using dirty water on the accuracy

**Figure 4:**
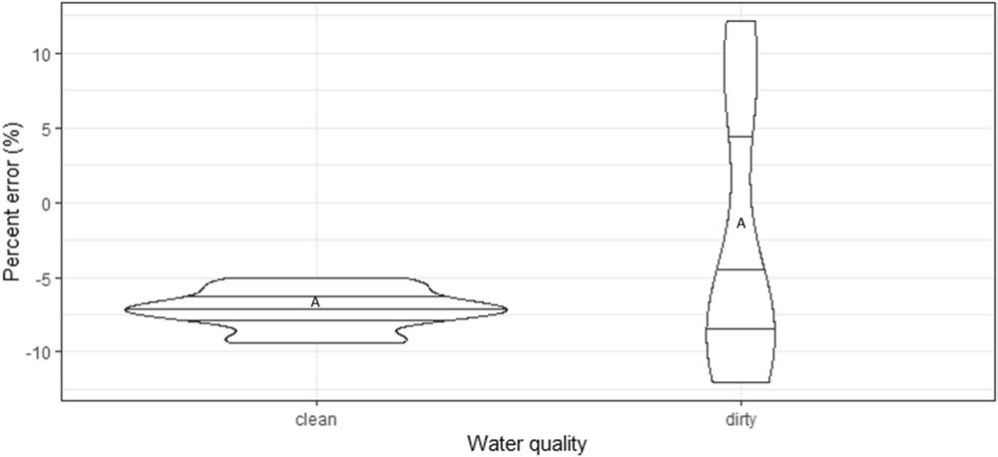
Relative accuracy (percent error) of the automated mosquito larval counter as a function of water types (clean and dirty water). Different letters indicate statistically different results between treatments.

When dirty water was used to count larvae, results showed a trend for reduced accuracy. The difference was not statistically significant (Fig. 4, df = 18, t = 1.81, *P* = 0.09). However, measurements were much more repeatable in clean than in dirty water (average of 1.32 and 3.90% respectively).

### Experiment 5: Assessing the effect of passing through the MLC sensor on larval survival, adult emergence and production percentages

Results for mosquito larval survival and development when passed or not passed through the MLC are summarized as follows: Larval mortality did not differ significantly between the control and the treatment (df = 3, *P* = 0.8) with an observed larval mortality of 1.90 ± 0.45% in control and 2.00 ± 0.73% in the treatment. Neither pupation percentage, 94.1 ± 1.03% in control and 94.8 ± 1.59% in the treatment, nor emergence percentage, 97.65 ± 0.91% and 96.64 ± 1.88%, respectively, were significantly different (df = 7, z = 0.69, *P* = 0.49 and df = 7, z = −1.34, *P* = 0.18 for pupation and emergence, respectively). Importantly, the number of larvae passed through the sensor that developed to adulthood (adult production percentage) was similar to the number of adults in the control (df = 7, z = −0.25, *P* = 0.81).

### Experiment 6: Comparative assessment of mosquito larval counter accuracy between Aedes aegypti, Aedes albopictus, and Anopheles arabiensis

**Figure 5:**
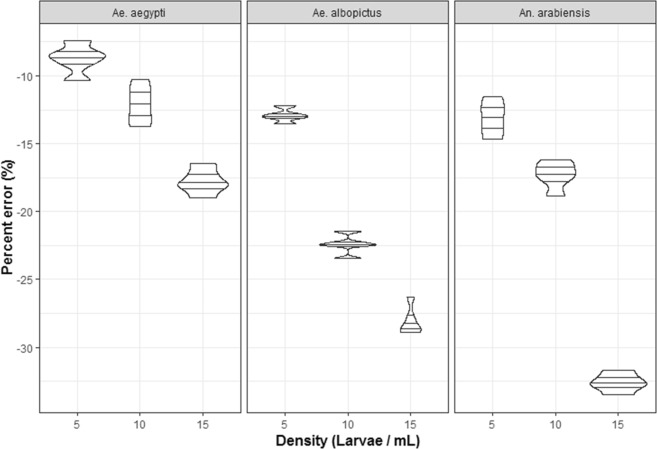
Relative accuracy (percent error) of the automated mosquito larval counter as a function of mosquito species (*Aedes aegypti, Ae. albopictus* and *Anopheles arabiensis*) using the same calibration.

Using the same calibration and the same sensitivity value (calibration made with *Ae. aegypti*, the sensitivity value was 0.700) for the three species, results showed that in all species, the accuracy significantly differed with density and mosquito species (Fig. 5, Table 2). At a fixed density and same calibration (sensitivity value set for *Ae. aegyti), Ae. albopictus* and *An. arabiensis* showed lower accuracies (e.g: for the larval density 5 one-day-old larvae/mL, the mean percent errors were −8.7, −12.9 and −13.1% for *Ae. aegypti, Ae. albopictus* and *An. arabiensis* respectively). However, when the calibration was performed independently for *Ae. albopictus* and *An. arabiensis* accuracies were −7.8 and −6.9% percent error respectively.

**Table 2:** Results of generalized linear mixed model for the effect of density and mosquito species on the accuracy of the mosquito larval counter.

	Estimate	SE	t value	Pr (>|t|)
(Intercept)	−8.755	0.463	−18.899	**<**2e-16
Density 10 larvae/mL	−3.437	0.655	−5.247	**7**.05e-06
Density 15 larvae/mL	−9.013	0.655	−13.757	**6**.63e-16
*Aedes albopictus*	−4.180	0.655	−6.380	**2**.16e-07
*Anopheles arabiensis*	−4.367	0.655	−6.666	**9**.05e-08
Density 10 larvae/mL: *Ae. albopictus*	−6.045	0.927	−6.524	**1.39e-07**
Density 15 larvae/mL: *Ae. albopictus*	−6.127	0.927	−6.613	**1.06e-07**
Density 10 larvae/mL: *An. arabiensis*	−0.737	0.927	−0.795	0.432
Density 15 larvae/mL: *An. arabiensis*	−10.457	0.927	−11.287	**2.22e-13**

Values were compared to the density 5 larvae/mL and *Aedes agypti*. Bold values are statistically significant.

## Discussion

The aim of this study was to validate the use of the MLC–CH1 for automatic mosquito larval counting. For any counting device, the accuracy and repeatability are basic requirements. In this paper, a mosquito larval counting device was evaluated using the two above mentioned parameters in relation to larval density, water type and mosquito larval age. Moreover, the impact of the device on larval survival and development was assessed. Overall, the results of this study demonstrated that the MLC-CH1 achieved a satisfactory level of accuracy and repeatability. However, a number of factors were shown to affect the accuracy of the MLC and therefore should be taken into account when performing automatic counting.

In these experiments, larval density affected the accuracy of the MLC. Higher larval densities increased the percent error absolute value and consequently decreased the accuracy. One possible explanation for this could be an uneven distribution of larvae in the input larvae container as suggested by Cringoli *et al*.^11^ when estimating the faecal egg counts of gastrointestinal strongyles and *Dicrocoelium dendriticum* in sheep. In fact, crowded larvae can result in overlapping larvae that are counted or measured as a single particle by the instrument. It is possible that the MLC-CH1 could not readily differentiate larvae in close proximity or that were overlapped. Further, it is necessary to calibrate each species and larval stage. The behavior, morphology, and size of mosquito larvae are species-specific, and different rearing protocols lead to further size variation. Because the optical sensor enumerates larvae based on size, a single calibration is not universally applicable. We believe, under recommended conditions, the MLC can be used for other mosquito species provided the calibration is performed prior to measurements. The repeatability is the closeness of multiple measurements values. Interestingly, our findings showed a lower level of variation between repeated measurements and did not find a significant difference over the range of the larval densities tested. A high level of repeatability, but low level of accuracy indicates systematic errors in the counting. As the repeatability is high and not greatly affected by some factors, the user can easily correct for the systematic error by subtracting the absolute value of percent error from the wanted number to achieve a high level of accuracy. For example, we found that when the larval densities were lower than 10 larvae/mL, the manually counting values were consistently 6 to 7% higher than the automated derived values. Due to the increase in true number of larvae, the channel limit value of the MLC should be set at 6% lower than the required number of larvae in the output container to compensate for the error. This greater repeatability of the MLC in addition to an appreciable time saving compared to the currently method in use at the IPCL represent a great advantage.

Water quality and the mosquito developmental stage are also factors that affect the accuracy of the MLC. The MLC performed well using clean water but poorly with dirty water. As the dirty water can contain some particles of food or other debris, this would likely be a source of error. The MLC provides the opportunity to count various mosquito ages (or stages). However, the mosquito developmental stage (eggs or larval age) affected the level of accuracy suggesting the importance of performing prior calibration for each mosquito species and larval age to be counted. In using this MLC, it is critical to properly separate first instar larvae or any other stage to be counted and make a good distribution in clean water. Although separating larvae from eggs shells using light/pipette adds an additional step to the process, this is a relatively simple task, and no negative impact was found in larval mortality when separating batches of 20,000 hatched eggs. However, we could not rule out the negative impact of high volume of larvae in small containers and care should be taken to prevent loss of larvae. This warrants further development methods for larvae/eggs separation and tests with high volumes of larvae. Future developments will also include a volumetric method based on the estimation of larval density in an aliquot of a large volume thereafter used to feed mass-rearing trays with a target number of larvae (18,000 larvae for *Aedes* mosquitoes in our references trays^12^.

Finally, it is important to note that larval survival, pupation success, adult emergence and production were not affected by the MLC. Passing through the sensor was not detrimental to survival or development of the immature stages.

## Conclusions

The results of this study demonstrated that the current MLC single channel prototype can be used efficiently to count mosquito larvae. The system provides repeatable and reproducible results. Despite its limitations, the error range is acceptable for our purposes, since the time burden is dramatically reduced. However, improved accuracy can be achieved with standardized procedure and careful work. Refinement (with a bigger larvae input container and a configuration with multiple channels) is ongoing, aiming to reduce the number of operations and ultimately speed up the process for potential use in mass rearing settings, which requires 18,000 first instar larvae per rearing tray. This technology could in future not only be used to fill first instar larvae in the trays to control the rearing density, but also to evaluate larval density in the tray before pupal collection as a quality control tool to estimate mortality.

## Methods

### Description and operating principle of the mosquito larval counter

**Figure 6:**
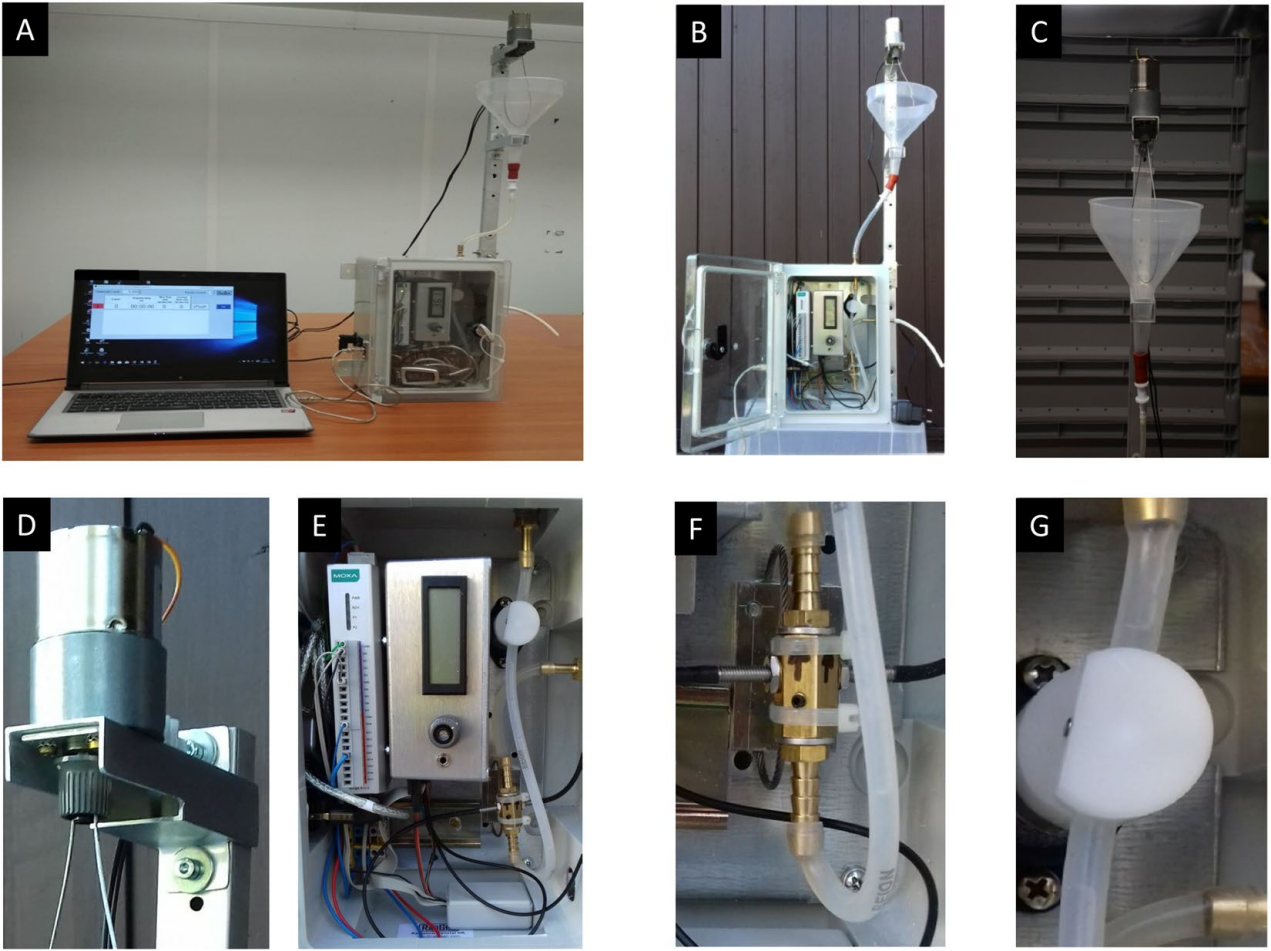
The automated mosquito larval counter. General view of the larval counter (**A**) general display of the electronic counting unit and the input larvae container (**B**) funnel as input larvae container (**C**) stirrer unit (**D**) electronic counting unit (**E**) optical sensor head (**F**) pinch valve (**G**).

The MLC-CH1 (Fig. 6) consists of 3 major parts: (i) an input larvae container (LC) or a funnel into which the sample is poured, a supporting frame and a stirrer unit, (ii) an electronic counting unit (ECU), (iii) the PC and the “Larvae” controlling program. A specially designed sensor head (Fig. 6F) is connected to an electronic fibre optic sensor unit (MLC-FO-01, Radiation General Ltd) by optical fibres. One optical fibre is connected to the light source output, another fibre is connected to the sensor input of the fibre optic sensor unit. The light beam in the sensor head passes perpendicularly through the water path, which is the so-called “Thrubeam” setting. Objects suspended in water passing by gravity at a controlled rate through the sensor head partially occlude the light beam which is converted by the fibre optic sensor unit to a voltage pulse which is proportional in amplitude to the particle size. If the decrease of the light intensity is greater than an adjustable threshold (the sensitivity set point), the passing object (larva) is counted by a data acquisition module (ioLogic-R1212, MOXA Ltd). A pich valve (P25-25, ChemDo Ltd) (Fig. 6G) controls the water flow and can be set to stop the flow at a set count. Result can be directly viewed in the software and the program records the measurement results (total number and time elapsed since start) in a log file.

### Source of mosquito colonies and maintenance

Experiments were performed using three established mosquito colonies maintained at the Insect Pest Control Laboratory (IPCL) of the joint Food and Agricultural Organisation/International Atomic Energy Agency (FAO/IAEA) Division of Nuclear Techniques in Food and Agriculture, Seibersdorf, Austria. The *Aedes aegypti* colony originated from Juazeiro, Brazil (provided by Moscamed, IAEA collaborative center) in 2012 and *Ae. Albopictus* originated from Rimini, Italy (provided by Centro Agricoltura Ambiente, IAEA Collaborative Center) in 2018. They were maintained under controlled temperature, RH and light regimes (26 ± 2 °C, 70 ± 10% RH, 11:1:11:1 hour light:dusk:dark:dawn photoperiod). Eggs used for these experiments were collected following the *Ae. aegypti* mass-rearing procedures developed at the IPCL^12–14^ and then stored in aluminium foil in 100 mL plastic cups. The *Anopheles arabiensis* colony (Dongola strain) originated from the Northern State of Sudan since 2005 and maintained under 27 ± 1 °C, 70 ± 10% RH, 11:1:11:1 hour light:dusk:dark:dawn photoperiod.

### Egg hatching and larvae separation

Dried eggs of *Aedes* mosquito species were gently brushed and hatched under laboratory conditions (28 ± 2 °C, 80 ± 5%) by submerging them overnight in cooled boiled osmosis water. Depending on the quantity of larvae needed for the experiments, either 700 mL water in sealable jars with 10 ml of larval food or 40 mL water in 50 ml falcon tubes with 2 mL larval food were used. For *An. arabiensis*, newly laid egg batches (<24 h old) were placed within floating plastic rings on the water surface of rearing trays for hatching.

Eggs, other stages or dust with a similar size could not be reliably differentiated. To avoid counting eggs, egg shells and irrelevant particles or debris leading to an overestimation and prevent delayed eggs hatching in larval trays, prior separation of 1^st^ instar larvae is a prerequisite. Twelve hours after hatching, larvae were separated from eggshells by placing a light source under the container, encouraging larvae to swim to the surface and away from the light where they were collected using a large pipette. After separation, the larvae were sieved through a 50 µm mesh and placed back in clean water for use.

### Calibration of the mosquito larval counter

The calibration was assessed by determining the appropriate value of the sensitivity following the calibration procedure given in the user manual provided by the manufacturer: info@rad-gen.com.

Experiments 1 to 5 were performed using *Ae. aegypti* only and experiment 6 using the three species *Ae. aegypti, Ae. albopictus* and *An. arabiensis*.

### Experiment 1: Determination of the accuracy of the mosquito larval counter and comparison to the current egg hatch rate-weighing method

In the preliminary step, three batches of 2000 first instar larvae were manually counted in three replicates. Automated counting was performed five times for each batch at a fixed larval density of 10 larvae/mL. The manual counts were performed by experienced trained operators. To determine the accuracy and validate the egg hatching-weighing method currently in use at the IPCL in mass rearing settings^4,12,14^, a second trial was performed as follows. Three sub-samples of around one hundred eggs (two weeks of age) were hatched overnight in 40 mL osmosis water with 2 mL larval diet using 50 mL falcon tubes. Following the protocol of Zheng *et al*.^4^, the mean hatch rate from the three sub-samples was used to estimate the number of eggs needed to obtain 1,000 first instar larvae. Fourteen samples of estimated egg numbers were weighted and hatched in 40 mL of cooled boiled deionized water overnight in sealed falcon tubes. The following day, all larvae were removed and separated from eggs shell using a light source and a pipette. Larvae were then manually counted prior to automatic counting (hereafter referred as input larvae count). Automated counting was performed once in 200 mL of osmosis water. To see if the number of larvae in the output container was the same as in the input count, manual counting was finally performed after automatic counts (hereafter referred as output larvae count). The accuracy and repeatability of the current method were calculated and the accuracy of the MLC relative to both input and output counts.

### Experiment 2: Assessing the effect of larval density on the accuracy and repeatability

Thirteen batches of 540 to 2700 first instar larvae were manually counted. Each batch of larvae was added to a container with osmosis water to get the following larval densities ranging from 2.2 to 23.3 larvae/mL. Larvae in suspension were transferred into the plastic funnel-shaped container of the MLC. Automated larval counting was then performed five times for each batch.

After this preliminary test, and to confirm or deny this observation, three larval densities (5, 10 and 15 larvae/mL) were selected for further analysis. Thus, 2,000, 4,000 and 6,000 first instar larvae were manually counted in two replicates each. Larvae were mixed in 400 mL of deionized water. Automated counting was repeated five times for each sample to evaluate the counter accuracy and within-run repeatability.

### Experiment 3: Assessing the possibility of using the mosquito larvae counter for different mosquito stages and ages

Mosquito stages and ages including eggs, one, two, three and four day-old larvae were tested for automatic counting by the MLC. In the first trial, the initial calibration performed for the first instar larvae as described above was used to estimate the accuracy of the MLC using different mosquito stages (from eggs to four day-old larvae). The same initial number of larvae was used for one, two, three and four-day-old larvae. Batches of 500 to 640 eggs and 544 to 584 one-day-old larvae were used in 250 mL of osmosis water for automated counting. Larvae were fed with 4% larval diet (after 24 h, one-day-old larvae were considered as 2nd instar and 48 h thereafter as 3rd instar and 72 h thereafter as 4th instar larvae).

One, three and four-day-old larvae were used to evaluate the effect of changing the value of the sensitivity on the accuracy. For each stage, the initial calibration was altered by adjusting the sensitivity multiple times to identify the value that gave the best accuracy.

### Experiment 4: Assessing the effect of using dirty water on the accuracy of the mosquito larval counter

It was hypothesized that small particles found in dirty water can be detected and counted by the MLC thereby decreasing its accuracy. To investigate this, larvae were reared in large mass-rearing trays (100 × 60 × 3 cm) containing 5 L of deionized water (18,000 first instar per tray). Twenty-four hours after the first pupae were observed in the rearing trays, the trays were tilted to collect the larvae and pupae and the rearing water called ‘dirty water’ collected. The ‘dirty’ water was passed through a 50-μm sieve (Retsch® Test Sieve with steel mesh) to discard large debris and water retained for use in this experiment. Two thousand first instar larvae were manually counted in three replicates and put in 200 mL of either clean or dirty water. Each sample was passed through the MLC sensor for automatic counting and the accuracy for both types of water calculated for comparison.

### Experiment 5: Assessing the effect of using the mosquito larvae counter on larval survival and development

To investigate if there was any detrimental effect of the MLC on larval mortality, pupation success, adult emergence and production, five batches of eggs, corresponding to 1,000 first instar larvae each (based on pre-determined hatch rates) were estimated following the procedures of Zheng *et al*.^3^. Eggs were distributed in each of five falcon tubes for hatching as described above. After egg hatch, 200 first instar larvae from each batch were manually counted (thereafter control). The remaining larvae from each batch were passed through the larval counter. After larvae were passed through the larval counter, 200 larvae were also manually counted (thereafter experimental). Larvae were reared to adulthood in plastic trays (13 × 6.5 × 5 cm) filled with 200 mL of deionized water and fed daily with 3 mL of 4% (wt/vol) larval diet. Larval survival after 24 h and 48 h was determined by manual counting the live larvae. At pupation, pupae were removed daily, counted and placed in individual emergence cages (17.5 × 17.5 × 17.5 cm, BugDorm-1H, MegaView, Taichung, Taiwan) until all adults emerged to determine emergence success.

### Experiment 6: Comparative assessment of mosquito larval counter accuracy between Aedes aegypti, Aedes albopictus, and Anopheles arabiensis

First instar larvae of the three species were tested for automatic counting by the MLC. We wanted to determine if the MLC can be used for all mosquito species using the same calibration or required a new calibration for each species. The calibration was first assessed using *Ae. aegypti* first instar larvae. The same calibration (same sensitivity value) was used to assess the accuracy. For each mosquito species, 2,000, 4,000 and 6,000 first instar larvae were manually counted. Larvae were mixed in 400 mL of deionized water. Automated counting was repeated five times for each sample and each species to evaluate the counter accuracy. Afterward, the calibration was done for *Ae. albopictus* and for *An. arabiensis* independently and the accuracy determined using 500 first instar larvae in 200 mL of clean water.

### Statistical analysis

The accuracy of measurements was quantified by calculating the percent error using the following formula: Accuracy (percent error) = ((manual count value − instrument count value)/manual count value) × 100 percent. The negative or positive sign indicate the direction of error from the manual count. Repeatability was quantified by calculating the percent of average value as: Repeatability (percent of average value) = ((difference between the highest and lowest values)/average count value) × 100 percent. All data were assessed using a series of linear models using R version 3.5.2 with the packages lattice, MuMIn, lme4, nlme and ggplot2^15^. Linear Gaussian mixed-effects models were fit by maximum likelihood (Laplace approximation) with the accuracy as response variable, density, larval stages, water type as fixed effects and the replicate as a random effect. In addition, binomial generalized linear mixed models with larval mortality, pupation and production, emergence rates as response variables and treatment as fixed effect and the replicate as a random effect were implemented.

## Data Availability

All data generated or analyzed during this study are included in this published article
